# Planting the Seeds: Orchestral Music Education as a Context for Fostering Growth Mindsets

**DOI:** 10.3389/fpsyg.2020.586749

**Published:** 2021-01-27

**Authors:** Steven J. Holochwost, Judith Hill Bose, Elizabeth Stuk, Eleanor D. Brown, Kate E. Anderson, Dennie Palmer Wolf

**Affiliations:** ^1^WolfBrown, Cambridge, MA, United States; ^2^Department of Psychology, Lehman College, City University of New York, New York, NY, United States; ^3^Longy School of Music of Bard College, Cambridge, MA, United States; ^4^Department of Psychology, West Chester University, West Chester, PA, United States

**Keywords:** music education, growth mindset, far transfer, socioemotional development, poverty, inequality

## Abstract

Growth mindset is an important aspect of children’s socioemotional development and is subject to change due to environmental influence. Orchestral music education may function as a fertile context in which to promote growth mindset; however, this education is not widely available to children facing economic hardship. This study examined whether participation in a program of orchestral music education was associated with higher levels of overall growth mindset and greater change in levels of musical growth mindset among children placed at risk by poverty. After at least 2 years of orchestral participation, students reported significantly higher levels of overall growth mindset than their peers; participating students also reported statistically significant increases in musical growth mindset regardless of the number of years that they were enrolled in orchestral music education. These findings have implications for future research into specific pedagogical practices that may promote growth mindset in the context of orchestral music education and more generally for future studies of the extra-musical benefits of high-quality music education.

## Introduction

Poverty and racism create gaps in opportunities for children to acquire the skills they need for success in school (Duncan and Brooks-Gunn, 2000). Recently, researchers have begun to explore the potential for growth mindset—the tendency to view one’s abilities as malleable rather than fixed (Dweck, 2009)—to serve as a protective factor that mitigates the effects of poverty and racism on school success (Claro et al., 2016). Students with a growth mindset tend to exhibit higher levels of school achievement over time, even after accounting for initial ability levels (Blackwell et al., 2007). This may be attributable, in part, to the fact that children with a growth mindset are more likely to respond to challenge with persistence and strategy shifting rather than giving up (Hong et al., 1999).

Persistence may be particularly important for children placed at risk by poverty and racism, given that these children are more likely to face challenges over the course of their development (Yoshikawa et al., 2012; Claro et al., 2016). Unfortunately, the risks associated with poverty and racism threaten not only opportunities for children to learn specific academic concepts (e.g., the scientific method) but also opportunities to develop the broader cognitive skills that undergird school success, including persistence in the face of challenge (Evans et al., 2005; Ackerman and Brown, 2010). Poverty-related instability and chaos, for example, have been linked to learned helplessness (Evans et al., 2005) and a lack of persistence in the face of challenge (Brown and Low, 2008). These threats to growth mindset underscore the importance of creating and sustaining different opportunities to support its development among children facing economic hardship. The present study examines the possibility that orchestral music education may promote growth mindset among children placed at risk due to poverty.

### Growth Mindset

The past 20 years have witnessed the growing acknowledgment of the importance of children’s socioemotional development (Zins et al., 2004), a broad term that includes decision making, interpersonal skills, and intrapersonal skills such as beliefs about oneself (Collaborative for Academic Social and Emotional Learning, 2019). These beliefs include the extent to which one’s own abilities are malleable, also referred to as one’s mindset. At one end of this continuum of mindsets is growth mindset or the view that one’s abilities can improve with effort and grow over time (Boylan et al., 2018); at the other end of the continuum is fixed mindset (Dweck et al., 1995) or the view that these abilities are stable (Dweck, 2009) and unlikely to improve with effort (Boylan et al., 2018).

Children who possess a growth mindset believe that the harder they work at something, the better they will be at it (Mrazek et al., 2018). Accordingly, they tend to choose more challenging tasks (Dweck and Leggett, 1988; Blackwell et al., 2007), view setbacks as opportunities to learn and improve (Davis, 2016; Burnette et al., 2018), and exhibit persistence and strategy shifting in the face of challenges (Hong et al., 1999). In contrast, children with a fixed mindset tend to choose easier tasks that allow them to demonstrate their current competence (Hong et al., 1999) and tend to give up more easily when confronted with challenges (Burnette et al., 2018). Not surprisingly, research suggests that, ultimately, children with a growth mindset academically outperform their peers who hold a fixed mindset, even when those children exhibit similar initial levels of achievement (Blackwell et al., 2007; Fraser, 2018).

Although mindset is sufficiently stable to be considered an aspect of personality, it is also dynamic across situations and malleable over time. Situational cues can lead children to adopt a growth orientation within a given context. Over time, repeated exposure to these cues can instantiate a growth mindset (Sternberg, 2005; Fraser, 2018). It is not surprising that the presence or absence of these cues in children’s school environments has been linked to individual differences in growth mindset (Dweck et al., 2014), given the amount of time children spend in educational environments. Extra-curricular activities have long been recognized as an important part of these environments (cf., Mahoney, 2000), and elements of different forms of extra-curricular arts education, in particular, may serve as an important context for the promotion of socioemotional development (Goldstein et al., 2017). One extra-curricular art education environment that may be an especially fertile ground for fostering children’s growth mindset is orchestral music education.

### Orchestral Music Education as a Context for the Development of Growth Mindset

The possibility that music education might promote extra-musical benefits is a controversial one. In general, the claims for the extra-artistic benefits of music and arts education have outpaced the evidence from rigorous research studies to support those claims (Hetland and Winner, 2001; Sala and Gobet, 2017). Many studies, for example, have employed strictly correlational designs, and the apparent relations between music education and extra-musical outcomes may be attributed to selection effects (e.g., smart children are more likely to pursue music lessons; Schellenberg, 2011), yet a growing body of evidence suggests that high-quality music and arts education may offer social–emotional benefits (Menzer, 2015) by training “habits of mind,” such as persistence in the face of challenge (Winner and Hetland, 2008). For example, in their “Studio Thinking Project,” Hetland and colleagues demonstrated that persistence was among the habits of mind potentially developed by intensive training in creating visual arts (Hetland et al., 2007).

Although no study of which we are aware has examined the possibility that orchestral music education may promote children’s growth mindset, the process of learning an instrument includes a number of structural features that have been found to promote a growth orientation, as does the process of learning an instrument in an orchestral context. As Hallam et al. (2012) noted, learning an instrument is not a single task but rather a series of successive tasks, each of which must be mastered according to a series of recursive steps. The student’s first step in mastering any of these tasks is to make an initial attempt: to try, for instance, to play a D-major scale for the first time. In doing so, the student identifies the *specific challenges* that they must overcome on route to mastery. For example, the first time they play a D-major scale, many students learning a woodwind instrument are likely to play a C-natural rather than a C# because in the G-major scale (the one they are most likely to have learned prior to D-major) C-natural should be played.

Having identified the challenge, the student must overcome it through the application of effort. The most straightforward approach is simple repetition, but it is also the least efficient (Hallam, 2001b). More experienced musicians employ more strategic approaches, such as focusing their practice on the aspect of the challenge that they find most difficult (Hallam, 2001a; Rotjan and Nicholson, 2020). As others have noted, in addition to setting challenges, one of the essential roles played by a music teacher is to scaffold these more strategic approaches to overcoming them (Rosenshine et al., 2002; McPherson, 2005). Having mastered the D-major scale, the student will turn to learning another major scale. A student learning a woodwind instrument will most often move in order to a scale with three sharps (A-major), then four (E-major), then five (B-major), and so on. The example of learning the major scales illustrates another structural feature of learning an instrument that may promote growth mindset: the challenges are *sequential* in that they occur in order of increasing difficulty, which allows children to monitor their progress toward mastery over time (Chronister, 2005; Pace and Lyke, 2011; Pike, 2013). They are also *open-ended* (Hallam et al., 2012): after learning the 12 major scales, the student will move on to learn the 12 natural minor scales, then the harmonic minor scales, then the melodic minor scales, and so on. As this example makes clear, mastering scales occurs over the course of years, and it is but one set of tasks entailed in learning an instrument.

In the context of orchestral music education, the conductor assumes many of the responsibilities of the teacher, setting specific and sequential challenges through the selection of repertoire and scaffolding strategic approaches by which the student ensemble can overcome those challenges (Goss, 2012; Alemán et al., 2017). The challenges remain open-ended—there is always more difficult repertoire to learn—but, in contrast to the individual pursuit of an instrument, they are also *collaborative* in that they must be overcome by groups of students working together in synchrony (Biasutti, 2013; Fasano et al., 2019). While group effort is, by definition, a feature of ensemble playing, its role is highlighted in the tradition of *Sistema*-inspired music education, which emphasizes the importance of every member of the ensemble to its collective success, regardless of their current level of proficiency (Alemán et al., 2017).

Prior research has linked each of these structural features of orchestral music education to improvements in children’s growth mindset. Presenting children with clear but appropriately challenging tasks may foster their growth mindset (Dweck et al., 2014); so too may sequential challenges that feature subsidiary goals against which a student can gauge their progress (Grant and Dweck, 2003). The open-ended nature of these challenges affords a practically inexhaustible supply that ensures another specific, sequential challenge will always be available. Prior research has also demonstrated that overcoming collective or corporative challenges is associated with higher levels of growth mindset (Matsui et al., 1987). One explanation for this association is that cooperative challenges may be more motivating for children (Johnson et al., 1981; Roseth et al., 2008). Another is that working cooperatively with peers may make goals seem more attainable than if those goals had to be pursued on one’s own (Matsui et al., 1987).

If we assume that orchestral music education can shift students’ mindsets to a growth orientation, a question remains about the domains in which that shift might be observed. The more conservative expectation would be that shifts in growth mindset would be confined to the context of the orchestra and perhaps musical endeavors more generally, that is, a student may increasingly come to see their abilities *as a musician* as subject to change. If experience with a particular music program led students to demonstrate greater growth mindset in terms of other musical endeavors, it would be an example of “near transfer.” A bolder expectation would be that gains in students’ musical growth mindset would prompt corresponding changes in students’ overall mindsets, such that orchestral music education could be said to have achieved “far transfer” where growth mindset was concerned (Barnett and Ceci, 2002). While this is an exciting possibility, the extant empirical literature attests to the difficulty of demonstrating far transfer of skills developed *via* music and arts programming (Hetland and Winner, 2001; Sala and Gobet, 2017).

### Access to Orchestral Music Education

Unfortunately, for many students in the United States, these questions are moot, given that the opportunity to learn to play an orchestral instrument is distributed unequally as a function of students’ income. Across the United States, less affluent students are more likely to attend schools where the positions of arts educators never existed (Abril and Gault, 2008) or have been eliminated (Parsad and Spiegelman, 2012), and it is these same students whose families are disproportionately unlikely to be able to purchase private instruction on the open market (Southgate and Roscigno, 2009; Duncan and Murnane, 2015). For lower-income families, the barriers to learning to play an *orchestral* instrument may be especially steep; purchasing or even renting a student model of a string instrument can cost hundreds of dollars, and borrowing instruments from a school is often only possible at the small set of schools serving less affluent families that nevertheless offer string instruction (Smith, 1997). As noted above, given the disproportionate challenges they face, growth mindset may be especially important for children in poverty. Therefore, if orchestral music education can indeed promote growth mindset, restricting access to that education on the basis of income may deny these children a particularly salient support for their development.

Many community music programs throughout the United States were founded, in part, to extend access to music education to less affluent students. More recently, programs inspired by Venezuela’s *El Sistema* model of orchestral music education have proliferated, and many of these programs have made providing orchestral music education to students who would otherwise not be able to afford it as a core component of their mission. A small number of studies have found that children enrolled in these programs realize benefits in a number of domains, including musical proficiency (Ilari et al., 2016), academic achievement (Holochwost et al., 2017), and executive functions (Holochwost et al., 2017; Sachs et al., 2017). However, no study of which we are aware has investigated whether participation in an *El Sistema*-inspired program of music education is associated with higher levels of growth mindset, and as such, no study has examined whether changes in musical growth mindset are domain-specific or whether they might transfer to general or overall growth mindset.

### Current Study

In the current study, we assessed growth mindset among students participating in one of 12 *El Sistema*-inspired programs of music education in the United States and their peers over the course of an academic year. We anticipated that students who were enrolled in an *El Sistema*-inspired program of orchestral music education would exhibit higher levels of *overall* growth mindset at the end of the academic year than their peers who were not enrolled in one of these programs. Moreover, we hypothesized that among students enrolled in an *El Sistema-*inspired program, levels of *musical* growth mindset (growth mindset in the domain of music) would increase significantly over the course of the program year.

## Materials and Methods

### Participants

Data were collected from a demographically diverse sample of 497 students (57% female) in the United States over the course of 2 consecutive academic years. Students in cohort 1 participated in the study during the 2015–2016 academic year, whereas students in cohort 2 participated in the following academic year (2016–2017). Therefore, each student’s participation in the study lasted for one academic year. At the time of their participation in the study, 62% of students had been enrolled in one of 12 *El Sistema*-inspired programs of music education for 1 (24%), 2 (17%), or 3 years (21%); comparison group students were recruited from the same schools and grades attended by the students in the programs. All students were in grades 3–5; the average age of the students was 10.2 years (*SD* = 1.05; see Table 1).

**TABLE 1:** Distribution of enrolled and unenrolled students by gender, ethnicity, and age.

	Overall (*N* = 497)	*M* (SD)	Unenrolled students (*N* = 190)	*M* (SD)	Enrolled students (*N* = 307)	*M* (SD)	Comparison	
							
	*n* (%)		*n* (%)		*n* (%)		*X*^2^ (*df*)	*t* (*df*)
**Gender**								
Female	280 (56.3)		97 (51.6)		183 (60.2)		3.51 (1)^T^	
Male	212 (42.7)		91 (48.4)		121 (39.8)			
Missing	5 (1.0)							
**Ethnicity**								
African American	130 (26.2)		49 (26.1)		81 (26.8)		23.0 (1)**	
Latino/Hispanic	179 (36.0)		61 (32.4)		118 (39.1)			
Asian/Pacific Islander	36 (7.2)		27 (14.4)		9 (3.0)			
American Indian	2 (0.4)		1 (0.5)		1 (0.3)			
Caucasian/White	38 (7.6)		12 (6.4)		26 (8.6)			
Other	56 (11.3)		21 (11.2)		35 (11.6)			
Mixed	49 (9.9)		17 (9.0)		32 (10.6)			
Missing	7 (1.4)							
Age		10.2 (1.05)		10.4 (0.98)		10.1 (1.09)		2.24 (455)*

For the enrolled and unenrolled student columns, the percentages are reported with respect to the number of students in each group and exclude students for whom information on gender or ethnicity was missing. ^*T*^p < 0.10, *p < 0.05, **p < 0.01.

Our memoranda of understanding with the *El Sistema-*inspired programs and their host schools prevented us from collecting individual-level socioeconomic data about the participating students’ families. However, all but two of the 12 programs included in the study had income eligibility guidelines that required most or all of the families they served to be of low income. Moreover, as shown in Table 1, over 90% of the sample were non-White, and over 60% of the participants were children of color (African American or Latino/Hispanic). In the United States, children of color are disproportionately likely to be from families living in poverty (Semega et al., 2020). Finally, all the of 12 programs in the study were located in areas with elevated levels of poverty. In 2019 (the most recent year for which data are available), the poverty rates in these areas ranged from 13.2 to 28.0%, with an average rate of 20.0%, which was nearly twice the national rate of 10.5% for the same year (Semega et al., 2020).

### Procedures

To be included in the study, *El Sistema-*inspired programs were required to offer instruction on an orchestral instrument to students in grades 3–5 (though they could serve students outside of this age range) and to offer that instruction weekly for a portion of the school year. The number of minutes of instruction per week and the number of weeks per year that instruction was offered varied, but on average, the programs offered 231.1 h of instruction per school year (*SD* = 105.1). All but two programs offered over 100 h of instruction per year, with hours of instruction offered by these programs ranging from 136 to 330. At each program, instruction was provided by teaching artists in a combination of small-group (string section) and large-group (orchestra) settings. One program, which was integrated into a music-focused charter school, offered instruction during the school day; the remaining programs took place after school.

At each program, a liaison was chosen in consultation with the study’s principal investigators and trained to lead data collection. Therefore, the liaison served in the role of a research assistant but was an employee of the program. The liaison was provided with a procedures manual as part of their training, which included a script to use when administering the measures. All students (those in one of the programs and those in the comparison group) completed the measure of overall growth mindset (see below) within 2 weeks of the beginning of the program year and again within 2 weeks of the end of the program year. Students enrolled in a program also completed the measure of musical growth mindset just after completing the overall growth mindset measure.

These procedures were approved by the Institutional Review Board of Bard College, of which the Longy School of Music is a part.

### Measures

Students’ overall growth mindset was assessed using a variant of the six-item measure developed by Dweck and colleagues, which has been demonstrated to exhibit both concurrent and predictive validity in numerous prior studies (Hong et al., 1999; Blackwell et al., 2007; Dweck, 2009). The measure was adapted to simplify the language, replacing terms such as “intelligence” with “smart” and “significantly” with “a lot,” and four additional items were added to the measure. All items were answered on a four-point scale (1 = not true, 4 = very true), and five items were worded so that lower scores were indicative of higher levels of growth mindset. Musical growth mindset was assessed using a parallel measure that replaced terms about intelligence with terms assessing musical aptitude. For example, the item from the overall growth mindset survey that read “You can learn new things, but you can’t really change how smart you are” became “You can learn new pieces of music, but you can’t really change how good you are at music.”

These measures were piloted in the academic year prior to the first cohort of students’ participation in the study, with a sample of 161 students recruited from 6 of the *El Sistema*-inspired programs that subsequently participated in the study. Students in the pilot study were of the same ages (mean age = 10.1 years, *SD* = 1.10 years) and similar demographic composition to those who completed in the full study: 59% of the pilot participants were female, and 22.2% were identified as African American, 42% as Latino/Hispanic, 6% as Asian/Pacific Islander, 10% as White, 7% as mixed, and 4% as other. The adapted overall measure of growth mindset displayed acceptable internal consistency (α = 0.83), and, although the mean was high (3.10), there was good variability about the mean (*SD* = 0.67) and minimal evidence of skew (*G*_1_ = −0.37, *SE* = 0.20). The measure of *musical* growth mindset, which was administered only to the subset of 72 students enrolled in an *El Sistema*-inspired program, displayed similar properties (α = 0.80, *M* = 3.14, *SD* = 0.62, *G*_1_ = −0.32, *SE* = 0.29). Scores on the overall and musical growth mindset measures were correlated [*r*(62) = 0.60, *p* < 0.001], suggesting that these two measures assessed related but distinct aspects of the common underlying construct of growth mindset.

Based on these pilot results, composite pre-program (α = 0.80) and post-program (α = 0.82) growth mindset scores were calculated as the mean of items (following reverse coding of appropriate items) for all students who answered at least 9 of the 10 questions. Parallel procedures were followed to calculate composite pre-program (α = 0.78) and post-program (α = 0.84) musical growth mindset scores. Composite overall and musical growth mindset scores were correlated at the pre-program [*r*(199) = 0.55, *p* < 0.001] and post-program [*r*(198) = 0.60, *p* < 0.001] assessments (see Table 2).

**TABLE 2:** Descriptive statistics for and bivariate correlations among age and pre- and post-program measures of overall growth mindset and musical growth mindset.

	1	2	3	4	5
1. Age	–				
2. Overall growth mindset, pre-program	0.16**	–			
3. Overall growth mindset, post-program	0.09	0.49**	–		
4. Musical growth mindset, pre-program	0.13*	0.55**	0.41**	–	
5. Musical growth mindset, post-program	–0.06	0.38**	0.60**	0.43**	–
*N*	457	427	441	251	254
*M*	10.2	3.06	3.11	3.17	3.25
SD	1.05	0.52	0.51	0.28	0.35

For pairwise correlations, N = (199, 426). *p < 0.05, **p < 0.01.

### Data Analysis

Data analysis was conducted in three steps. In the first preliminary step of data analysis, we examined: (1) whether pre-program overall growth mindset scores differed by group, (2) the distribution of post-program overall growth mindset scores, (3) the relation between demographic characteristics (gender, ethnicity, and age) and post-program measures of overall and musical growth mindset, and (4) the patterns of missingness for the growth mindset measures. This final stage in the preliminary analyses was accomplished by regressing missingness for the post-program measures of overall and musical growth mindset (coded dichotomously) on demographic characteristics, program enrollment, or pre-program measures of growth mindset (see Jelicic et al., 2009). In the second step, we specified and tested a multilevel linear model in which post-program levels of overall growth mindset were estimated as a function of the number of years students had been enrolled in the program while controlling for pre-program overall growth mindset and relevant covariates. This model accounted for the hierarchical structure of the data, in which students were nested within program sites. In the third and final step of analysis, we specified and tested a multilevel linear model that examined whether *musical* growth mindset scores changed over time. In this model, time of measurement (pre-program and post-program) was nested within student, while students were nested within program sites, and the focal predictor was time rather than the number of years students were in the program.

## Results

### Preliminary Analyses

Table 2 displays descriptive statistics for and bivariate correlations among student age and scores on overall and musical growth mindset measures administered prior to and following the program. Students enrolled in a program exhibited slightly higher pre-program overall growth mindset scores. The magnitude of this difference was equivalent to 0.08 SD (*M*_diff_ = 0.04/*SD* = 0.52) and therefore required that pre-program scores be included in subsequent models to satisfy the requirements of baseline equivalence. Post-program overall growth mindset scores were distributed in an approximately normal fashion, though there was some evidence of negative skew that was not attributable to outliers (*G*_1_ = −0.42, *SE* = 0.12). A similar distribution was observed for post-program musical growth mindset scores (*G*_1_ = −0.55, *SE* = 0.15), though in this case one outlying score was Winsorized to the next lowest score that was not an outlier.

While gender was not related to post-program overall (*p* = 0.643) or musical growth mindset scores (*p* = 0.790), ethnicity was related to overall growth mindset scores [*F*(6, 434) = 5.88, *p* < 0.001]. African American students reported significantly lower levels of overall growth mindset than their peers identifying as Latino/Hispanic or Asian/Pacific Islanders. Missingness of post-program musical growth mindset scores was not related to gender, ethnicity, age, program enrollment, or pre-program musical growth mindset scores. Missingness of post-program musical growth mindset scores was related to age [Wald(1) = 7.05, *p* = 0.008], such that older students were more likely to be missing these scores. Therefore gender, ethnicity, and age were included as covariates in models estimating overall growth mindset, given that the distribution of enrolled and unenrolled students differed by gender (at a level approaching significance), ethnicity, and age and that ethnicity was related to overall growth mindset scores. Age was included as a covariate in the model estimating musical growth mindset.

### Model Specification and Testing

#### Overall Growth Mindset

A random-effects ANOVA was estimated to partition the variance in post-program growth mindset scores into portions attributable to program factors (2.9%, *p* = 0.112) and child factors (97.1%, *p* < 0.001). Post-program overall growth mindset scores were estimated for the *i*th child in the *j*th program according to the following equation:

(1) $$p\,o\,s\,t\,\text{-}\,p\,r\,o\,g\,r\,a\,m\,g\,r\,o\,w\,t\,h\,m\,i\,n\,d\,s\,e\,t_{\text{ij}}=i\,n\,t\,e\,r\,c\,e\,p\,t+p\,r\,e\,\text{-}\,p\,r\,o\,g\,r\,a\,m\,g\,r\,o\,w\,t\,h\,m\,i\,n\,d\,s\,e\,t_{\text{ij}}+g\,e\,n\,d\,e\,r_{\text{ij}}+e\,t\,h\,n\,i\,c\,i\,t\,y_{\text{ij}}+a\,g\,e_{\text{ij}}+y\,e\,a\,r\,s_{\text{ij}}+e\,r\,r\,o\,r_{\text{ij}}$$

wherein pre-program growth mindset is a continuous variable and gender, ethnicity, and years are categorical variables. The variable years had four levels, corresponding to the number of years a student had been enrolled in the program (with a value of zero corresponding to students who were not enrolled in the program).

**FIGURE 1:**
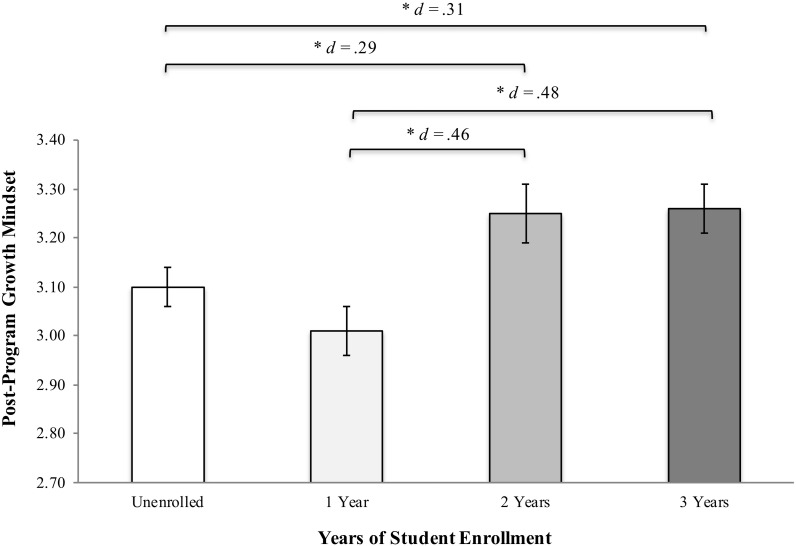
Post-program overall growth mindset scores by years of student enrollment. Note that the brackets indicate significant Tukey-adjusted contrasts (^∗^*p* < 0.05), and *d* values correspond to effect sizes calculated by dividing the difference in the model-implied estimates for each pair of groups by the pooled standard deviation of pre-program overall growth mindset scores.

The specific contrast between students who were not enrolled in the program (years = 0) and those who were [years = (1, 2, 3)] was not significant (*p* = 0.900), indicating that there was no difference in post-program overall growth mindset scores between students who were enrolled in the program and those who were not. However, the omnibus test of years (i.e., the type III test of fixed effects) indicated that there were significant differences in post-program growth mindset scores as a function of students’ years of program enrollment [*F*(3, 376) = 3.10, *p* = 0.027]. Tukey-adjusted contrasts indicated that, although there was no significant difference in post-program growth mindset scores between students who had been enrolled in the program for 1 year and those who had not been enrolled in the program, students enrolled in the program for 2 years exhibited significantly higher post-program growth mindset scores than students enrolled for 1 year or no years. Similar differences were observed between students who had been enrolled for 3 years and those enrolled for 1 year or no years (see Figure 1).

#### Musical Growth Mindset

Given that measures of musical growth mindset were collected only from students enrolled in a program, our model was parameterized so that the *i*th time of measurement was nested within the *j*th student, who was nested within the *k*th program. A random-effects ANOVA was estimated to partition the variance in post-program growth mindset scores into within-child (42%, *p* < 0.001), between-child (54%, *p* < 0.001), and between-program (3.9%, *p* = 0.064) portions. Musical growth mindset scores were estimated for the *i*th timepoint for the *j*th student enrolled in the *k*th program according to the following equation:

(2) $$g\,r\,o\,w\,t\,h\,m\,i\,n\,d\,s\,e\,t_{\text{ijk}}=i\,n\,t\,e\,r\,c\,e\,p\,t+t\,i\,m\,e_{\text{i}}+a\,g\,e_{\text{ijk}}+e\,r\,r\,o\,r_{\text{ijk}}$$

wherein time is a categorical variable with two levels: 0 = pre-program and 1 = post-program.

The parameter estimate for time was statistically significant and positive [*B* = 0.09, *t*(272) = 3.02, *p* = 0.003], indicating that musical growth mindset scores increased over the course of the program year. Model-implied estimates indicated that, on average, pre-program score was 3.16 (*SE* = 0.03), while post-program score was 3.25 (*SE* = 0.03), corresponding to an effect size of *d* = 0.32. To examine whether the degree of residualized change in musical growth mindset scores varied as a function of the number of years students had been enrolled in the program, the model specified in Eq. 2 was re-estimated after introducing the interaction between time and years. As in Eq. 1, the variable years was a categorical variable but, in this case, had only three levels, given that comparison-group students did not complete the musical growth mindset measure. The parameter estimate for the interaction term was not significant (*p* = 0.190).

## Discussion

The prospect that orchestral music education might promote growth mindset is both exciting and controversial. It is exciting because growth mindset matters for all children and particularly for those who face the risks associated with poverty and racism that inculcate cognitive (Bolland, 2003) and neurophysiological (Evans, 2003) patterns of learned helplessness. However, it is also controversial because claims about the ancillary benefits of music and arts education have often failed the test of rigorous empirical research (Hetland and Winner, 2001; Sala and Gobet, 2017). The present study was designed as a robust test of whether participation in an *El Sistema*-inspired program of orchestral music education might predict significantly higher levels of overall growth mindset and increases in musical growth mindset among children at risk.

As hypothesized, we found that participation in an *El Sistema-*inspired program of orchestral music education was associated with higher levels of overall growth mindset. After controlling for key demographic variables and initial growth mindset scores, students participating in a program showed higher levels of growth mindset than their peers who did not participate. Although this finding is consistent with a growing body of evidence that high-quality and intensive music education programs may be linked to extra-musical benefits (Menzer, 2015), this is the first study of which we are aware to reveal an association between music program participation and enhanced growth mindset. Furthermore, within this overall association, we observed a dose–response effect: Only students who had been enrolled in the *El Sistema-*inspired program for 2 or 3 years exhibited higher year-end levels of overall growth mindset than their peers who did not participate. The scores of students who had been enrolled for a single year were not significantly different from those of the comparison group. This is consistent with findings suggesting that certain extra-musical benefits of music and arts programming may emerge over time and with accumulated exposure [see, for example, Brown et al. (2017)].

Our analyses also supported the hypothesis that participation in the *El Sistema-*inspired program would be associated with higher levels of musical growth mindset. In fact, students in the program for 1 year *did* exhibit significant increases in their *musical* growth mindset as did students who were in the program for 2 or 3 years. Moreover, bivariate correlations suggested that musical growth mindset was associated with overall growth mindset. This pattern of results suggests an intriguing possibility: for the reasons outlined above, orchestral music education may be a fertile context for the development of growth mindset. However, the rate at which these changes take root may differ by domain. Orchestral music education may first affect changes in musical growth mindset, achieving near transfer to a domain that is proximal to the experience of music education itself. Far transfer to the more distal domain of overall growth mindset may take longer to occur. Whereas, the literature on the extra-musical benefits of music education has often distinguished between the reasonable possibility of near-transfer and more unlikely possibility of far transfer (e.g., Sala and Gobet, 2017), the present findings suggest that this distinction may be at least, in part, an artifact of dose. The fact that students in the program for 1 year demonstrated increases in musical growth mindset but those in the program for 2 or 3 years demonstrated higher levels of overall growth mindset suggests that near-transfer may precede far transfer and that the latter may indeed be attainable upon sufficient dose.

### Limitations and Directions for Future Research

As appealing as this possibility is, it is at this point just that—a possibility. In order to examine whether change in musical growth mindset predicted subsequent change in overall growth mindset, a longitudinal design spanning multiple years would be required. This design would also reduce the potential for differential attrition to bias our results with respect to overall growth mindset. The finding that students who were in the program for 2 or 3 years exhibited higher levels of overall growth mindset at year-end may be attributable, in part, to the possibility that students who remain in the program for 2 or 3 years have higher levels of growth mindset *before* beginning musical instruction. While we controlled for levels of growth mindset collected at the beginning of the program year, for students in the program for 2 or 3 years, this data point did not coincide with their levels of overall growth mindset prior to enrolling in the program.

This possibility underscores one limitation of our study: the inability to draw causal inference. Students in our study (or possibly their families) self-selected to participate in an *El Sistema*-inspired program. It is, therefore, possible that students with higher levels of overall growth mindset chose to pursue musical study or that parents who engage in behaviors that are more likely to foster growth mindset chose to have their children participate in a program. Two features of the present study—its quasi-experimental design and the fact that we controlled for initial levels of overall growth mindset—minimize this possibility but cannot eliminate it. Therefore, a longitudinal design that incorporated random assignment would be the preferred design to employ in future research, and to the extent that this design included an active control condition (in which children were engaged in a set of other structured activities), it would allow for the possibility of isolating the specific effect of music education on growth mindset.

While not a limitation *per se*, it is also important to note that our sample was comprised entirely of students from the United States. This means that key components of the context in which the *El Sistema*-inspired programs included in the study operate may not be found in other countries. For example, although extra-curricular arts education programs are at least partially subsidized by the government in much of the developed world, in the United States, they are funded primarily through donations from individuals, corporations, and private foundations. As a result, disruptions in revenues due to economic events (such as the 2008 recession) can undermine the capacity of these programs to provide continuous instruction. Thus, the context in which orchestral music education occurs in the United States may differ from the context in which it occurs in other countries, just as the broader context in which development unfolds differs. These differences may together restrict the generalizability of the findings reported here to children raised in other countries and contexts.

The final limitation of the present study is its emphasis on the structural, rather than the pedagogical, features of orchestral music education. As we noted above, all the programs included in our study shared certain structural features, such as a combination of small- and large-group instruction. Some of those structural features may foster students’ growth mindset, and some of those features—such as placing students in orchestras at an earlier point in their musical development than is generally the case and emphasizing sectional and full-ensemble rehearsals over private instruction—may be particularly effective in fostering growth mindset by offering opportunities to pursue corporative goals. However, at this point, emphasis must be placed on *may*—while these structural features of orchestral instruction may imbue it with the potential to foster growth mindset, whether that potential is realized is undoubtedly influenced by the approach to teaching employed by a particular program or instructor. All else being equal, a program that instilled an ethos of cooperation and ensemble achievement among its students would be more likely to maximize opportunities to overcome corporative goals than a program that encouraged competition and focused on the success of some students to the exclusion of others.

Perhaps the most important aspect of pedagogy as it relates to growth mindset is the nature of the feedback teachers provide to students. Making mistakes is an essential part of learning an orchestral instrument. If a teacher works with a student to acknowledge and reflect on their mistakes in way that focuses on the potential for improvement, it can foster that student’s growth mindset (Davis, 2016), but it is just as important that a teacher is mindful of the praise they provide to a student when they perform well. In a seminal study by Kamins and Dweck (1999), generic, person-focused praise (e.g., “You are a good student”) following success fostered helplessness in response to a subsequent mistake. Therefore, praise that emphasizes innate ability or talent is a double-edged sword: while the student may welcome being told that they are “a natural” when they perform well, when they inevitably struggle, they may wonder if that talent has somehow evaporated (Davis, 2016). This, in turn, may make a student wary of trying more challenging pieces or excerpts, ultimately inhibiting their progress as a young musician (Mueller and Dweck, 1998; Kamins and Dweck, 1999; Cimpian et al., 2007). For these reasons, praise that emphasizes effort and persistence is more likely to foster a growth mindset (Cogdill, 2014). Given the relation between teacher feedback and students’ growth mindset, it is very likely that the association between orchestral music instruction and growth mindset is moderated by teacher behavior (and by the behavior of the conductor as well).

Therefore, a clear direction for future research is to observe and codify teacher behaviors so that their interaction with the context of orchestral music education in promoting growth mindset can be better understood, a direction we have begun to pursue. In a recent round of data collection, we were able to observe and code teacher behaviors at two of the programs included in the study reported here, programs that are widely acknowledged as leaders within the field of *Sistema*-inspired orchestral music education. These observations revealed that teachers’ feedback to students featured high levels of language about the value of effort and persistence (Wolf et al., 2019). Perhaps not coincidentally, students who attended these programs exhibited some of the largest gains in both musical and overall growth mindset, a preliminary finding that speaks to the importance of conducting research into the prevalence of pedagogical practices known to foster growth mindset in the context of orchestral music education.

## Conclusion

In spite of its limitations, this study makes a contribution to literature. It is the first to demonstrate an association between orchestral music education and growth mindset. The fact that this association was found in a sample of under-served students raises the intriguing prospect of incorporating elements of programs explicitly designed to boost growth mindset among students placed at disadvantage by poverty (e.g., Blackwell et al., 2007; Yeager et al., 2016; Burnette et al., 2018) into the design of *El Sistema*-inspired programs to maximize their potential effects. Although at present it is not possible to infer causation or claim that orchestral music education has a unique capacity to promote growth mindset, the results reported here demonstrate the potential for orchestral music education to foster growth mindset, a potential that future studies should more fully explore.

## Data Availability Statement

The datasets for this article are not publicly available because data were collected from minors and could be identifiable using certain demographic information given the relatively small number of participants per site. Requests to access the datasets should be directed to SH, steven.holochwost88@login.cuny.edu.

## Ethics Statement

The studies involving human participants were reviewed and approved by the Institutional Review Board of Bard College and IntegReview, a private Institutional Review Board. Written informed consent to participate in this study was provided by the participants’ legal guardian/next of kin. Written informed consent was not obtained from the minor(s)’ legal guardian/next of kin for the publication of any potentially identifiable images or data included in this article.

## Author Contributions

SH, JB, and DW designed the study. ES oversaw the data entry and management and assisted SH with the statistical analyses. SH prepared the initial draft of the manuscript, to which all authors subsequently made contributions.

## Conflict of Interest

The authors declare that the research was conducted in the absence of any commercial or financial relationships that could be construed as a potential conflict of interest.
